# Response of sediment delivery ratio to water-sediment and riverbed boundary conditions during flood events in the lower yellow river since 2000

**DOI:** 10.1038/s41598-026-42616-7

**Published:** 2026-03-07

**Authors:** Xueqin Zhang, Min Zhang, Chunjin Zhang, Xiaohua Zhang, Zanying Sun

**Affiliations:** 1 Institute of Geographic Sciences and Natural Resources Research, Chinese Academy of Sciences, Beijing, 100101 China; 2 University of the Chinese Academy of Sciences, Beijing, 100049 China; 3 Yellow River Institute of Hydraulic Research, Yellow River Conservancy Commission, Zhengzhou, 450003 China; 4 Key Laboratory of Lower Yellow River Channel and Estuary Regulation, Ministry of Water Resources, Zhengzhou, 450003 China

**Keywords:** Sediment transport, Sediment delivery ratio, Water-sediment conditions, Riverbed boundary, Flood events, Lower Yellow River, Environmental sciences, Hydrology

## Abstract

The sediment delivery ratio is greatly affected by the water-sediment and the riverbed boundary, which represent the river’s capacity to transport sediment under specified conditions. This study examines the response of the sediment delivery ratio to water-sediment and riverbed boundary conditions in the Lower Yellow River (LYR) since the operation of the Xiaolangdi Reservoir began. It evaluates the spatial-temporal variations of water-sediment and riverbed boundaries based on hydrological data and topographic data from 2000 to 2023. Based on the sediment transport rate equation, a theoretical equation for the sediment delivery ratio during flood events has been developed, thoroughly considering the effects of riverbed boundary conditions, including median particle size of bed sediment, river gradient, and width-to-depth ratio. The results show that the sediment delivery ratio negatively correlates with the incoming sediment coefficient, the median particle size of bed sediment, and the width-to-depth ratio. In contrast, it positively correlates with the water load variation coefficient and river gradient. In comparison to solely accounting for water and sediment conditions, incorporating the riverbed boundary into the theoretical equation results in a more precise alignment with the measured sediment delivery ratio data. This indicates that the riverbed boundary is a crucial factor influencing the sediment delivery ratio. Under the current boundary conditions, the Aishan to Lijin reach has the highest sediment transport capacity. To improve the sediment transport capacity of the Tiexie to Lijin reach, it is recommended to narrow the river width upstream of Gaocun and increase the width-to-depth ratio. The findings of this study provide essential scientific insights into the sediment transport capacity of alluvial rivers subjected to variations in water-sediment and riverbed boundary conditions, thereby offering important references for hydrological engineering and river management practices.

## Introduction

Rivers are essential resources for human civilization and economic growth, playing a critical role in the global ecosystem, the carbon cycle, and the propagation of life^1–3^. River sediment transport is essential in river dynamics, affecting the feedback loop between water, sediment, and channel cross-section shape^4–6^. The sediment delivery ratio serves as a crucial indicator that illustrates the dynamics of sediment transport within a river channel. It delineates the proportion of sediment load at the outlet relative to that at the inlet over a specified period.

Since the inception of human civilization, societies have preserved a history of survival and progress along riverine regions. They have advanced and utilized river runoff management—primarily through reservoir construction—to mitigate the impacts of floods and droughts. Since the 1950 s, large−scale reservoir construction has been ongoing globally. Currently, more than 45,000 large reservoirs (exceeding 15 m in height) have been constructed, primarily distributed across 140 countries^7,8^. The construction of reservoirs has modified downstream runoff and sediment transport, disturbed the natural hydrological equilibrium, and resulted in the reconstruction of the downstream riverbed^9–11^. This has resulted in various degrees of alteration in sediment transport features, especially in the Mississippi River, Colorado River, Yellow River, and Yangtze River^9,12–22^. Since the Xiaolangdi Reservoir (China) began operating for runoff storage and sediment interception in October 1999, there has been a sharp decrease in the sediment load entering the downstream channel. This has resulted in ongoing erosion of the downstream riverbed and substantial alterations in sediment transport capacity^23–28^.

In recent years, many scholars have utilized the sediment delivery ratio concept to investigate the sediment transport characteristics of flood events in the Lower Yellow River (LYR). Historically, two conventional approaches have been employed to ascertain the theoretical equation of the sediment delivery ratio. One approach involves analyzing the influencing factors and subsequently establishing the theoretical equation through direct statistical regression utilizing the measured sediment delivery ratio^29–39^. An alternative approach entails deriving the theoretical framework of the sediment delivery ratio through the sediment transport rate relationship, subsequently calibrating the parameters with measured data to develop the theoretical Eqs^36,40–44^. The above results analyzed how water and sediment conditions influence the sediment delivery ratio across different temporal and spatial scales before 2000. It was also suggested that factors such as the incoming sediment coefficient, average sediment concentration, and water load variation coefficient serve as key determinants of the sediment delivery ratio in downstream rivers.

These applications have provided valuable insights into the relationship between sediment delivery ratio and water-sediment conditions. In recent years, due to climate change and human activities, many rivers in China have exhibited significant changes in water and sediment levels^45,46^, resulting in notable adjustments to riverbed morphology^47^. However, previous research has overlooked how riverbed boundary conditions influence sediment transport adjustments in fluvial dynamics, which has limited our understanding of the physical processes that affect changes in sediment delivery ratios in river systems^47^.

This paper evaluates the spatial-temporal variations of water-sediment and riverbed boundary in the LYR since the commencement of operations at the Xiaolangdi Reservoir. Based on the sediment transport rate equation, a theoretical equation for the sediment delivery ratio during flood events is developed, which thoroughly accounts for the effects of riverbed boundary conditions, including median particle size of bed sediment, river gradient, and width-to-depth ratio. Considering the current riverbed boundary conditions, the sediment transport characteristics across various reaches in the LYR are elucidated, thereby offering a theoretical foundation for comprehending the comprehensive influence of water-sediment interactions and riverbed boundary conditions on sediment transport mechanisms.

## Study area, data, and methods

### Study area

**Fig. 1:**
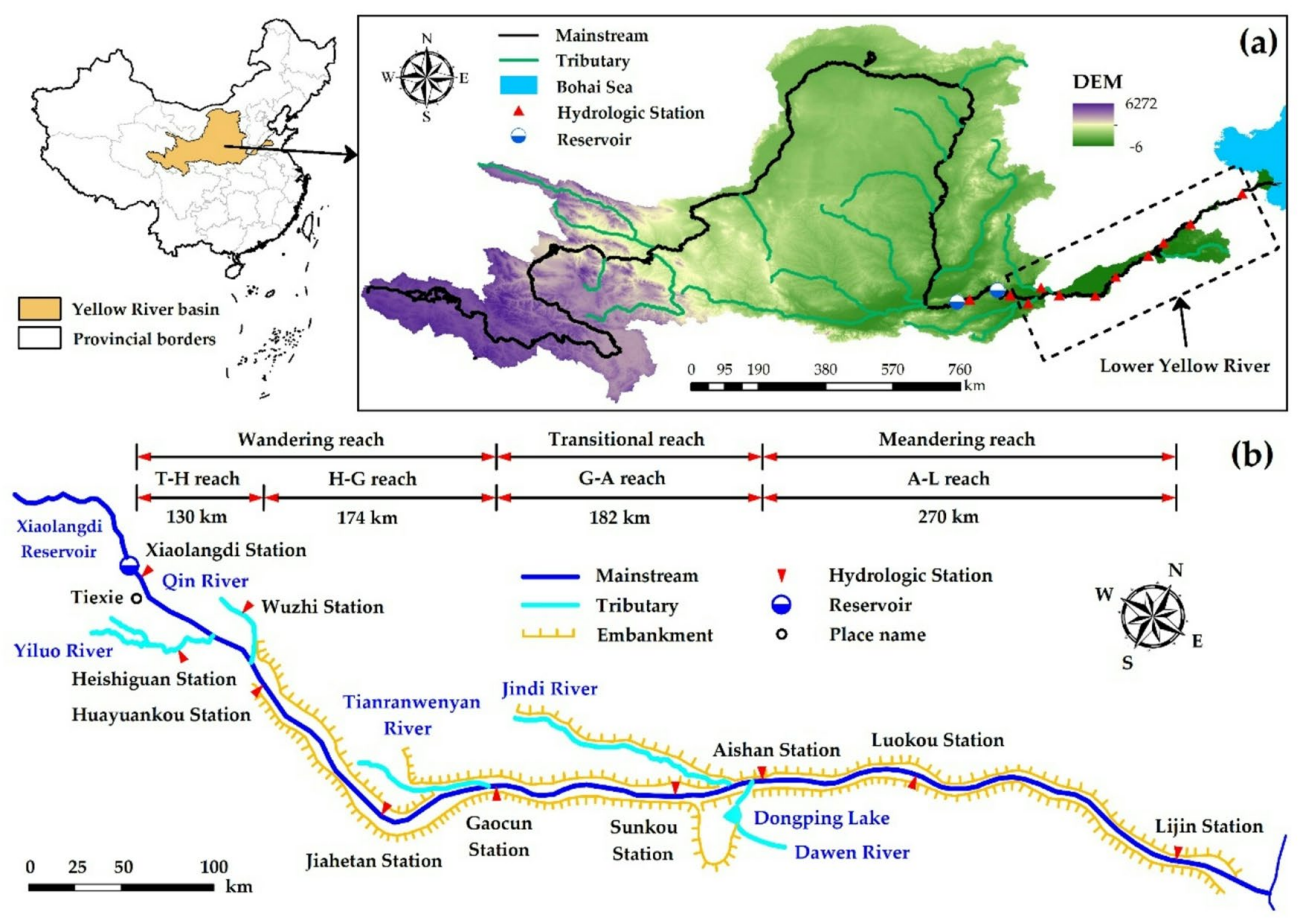
Schematic diagram of the LYR. **(a)** Yellow River basin. **(b)** Lower Yellow River. The map was generated by the authors using ArcGIS 10.8 (http://www.esri.com/software/arcgis) and does not require any permissions.

The Yellow River is the second−longest river in China. The LYR begins at Tiexie (China) located downstream of the Xiaolangdi Reservoir. Subsequently, two major tributaries converge into the mainstream river: the Yiluo River and the Qin River, traversing the North China Plain before ultimately discharging into the Bohai Sea near Lijin. Due to long−term sedimentation, the riverbed in the LYR gradually rises, forming a “Hanging River” that is higher than the ground on both sides. Figure 1is a schematic diagram of the LYR. The total length of the LYR is approximately 756 km, and the river’s cross-sectional profile is wide upstream and narrow downstream, with the slope of the riverbed steep upstream and gentle downstream^48,49^. Based on the river’s morphology and channel dynamics, the LYR can be categorized into wandering, transitional, and meandering reaches, namely, Tiexie to Gaocun is a wandering river reach, Gaocun to Aishan is a transitional river reach, and Aishan to Lijin is a meandering river reach^50,51^. There are a total of seven hydrological stations along the LYR: Huayuankou, Jiahetan, Gaocun, Sunkou, Aishan, Luokou, and Lijin (China). The LYR studied in this article refers to the river reach from Tiexie to Lijin (T − L reach). Based on river sedimentation, the LYR is divided into four reaches: Tiexie to Huayuankou (T − H reach), Huayuankou to Gaocun (H − G reach), Gaocun to Aishan (G − A reach), and Aishan to Lijin (A − L reach), as illustrated in Fig. 1. The map in Fig. 1 was prepared by the co-authors using ArcGIS 10.8 (http://www.esri.com/software/arcgis).

The Xiaolangdi Reservoir is located 130 km downstream of the Sanmenxia Reservoir (China) and 128 km upstream from the Huayuankou Station, with a total storage of 126.5 × 10^8^ m^3^ and sediment trapping storage of 75.5 × 10^8^ m^3^^52^. The operation of the Xiaolangdi Reservoir in October 1999significantly changed the downstream water-sediment relationship, especially after water-sediment regulation began in 2002^53^.

### Data

Data on hydrological data of 159 flood events were collected from eight hydrological stations (Xiaolangdi, Huayuankou, Jiahetan, Gaocun, Sunkou, Aishan, Luokou, and Lijin) and two tributary stations (Heishiguan and Wuzhi) from 2000 to 2023, including flow discharge, sediment concentration, incoming sediment coefficient, and water load variation coefficient, as reported in the “*Hydrological Data of the Yellow River Basin*”^54^, “*Yellow River Sediment Bulletin*”^55^, and “*Yellow River Water Resources Bulletin*” ^**56**^. Hydrological measurements in the Yellow River Basin have been conducted by the Yellow River Conservancy Commission since the 1950s^24^. According to statistics, there are a total of 340 large cross-sections in the LYR. The repeated surveys of cross-sectional profiles have been conducted twice a year, before and after the flood seasons from July to October at each sedimentation section, which is located at a fixed site. To investigate the channel evolution characteristics in the LYR, 46 sets of cross-sectional terrain data were collected, including bankfull width, bankfull depth, bankfull width-to-depth ratio, and median particle size of bed sediment.

The selection criteria for flood events in this paper exclude long-duration, low-flow processes and focus on floods with significant fluctuations and an average flow discharge exceeding 500 m³/s^36,44^. When the average flow discharge of the flood event is below 500 m^3^/s, the flood process has a weak sediment transport effect, and there are no flood events with high sediment concentration. The classification criteria for flood events adhere to the principles established in the “*Documented data of fluvial processes of the Lower Yellow River*”^57^. The selected flood events can represent various combinations of water and sediment, and the theoretical equation of sediment delivery ratio established with this data has broad applicability^58^.

## Methods

### Calculation of sedimentation

According to the flood propagation process along the channel, after the flood discharge process of the Xiaolangdi Reservoir, Huayuankou Station, Gaocun Station, Aishan Station, and Lijin Station implemented respective flood processes for 1d, 2 d, 4 d, and 5 d^59^. This approach effectively addressed water addition and diversion from tributaries to maintain the integrity of water and sediment propagation.

The sediment transport rate method is employed to calculate the sedimentation of each reach^60^:

1 $$\vartriangle {W_s}={W_{s,in}}-{W_{s,out}}-{W_{s,d}}$$

where *∆W*_*s*_ is the sedimentation load, 10^8^ t; *W*_*s, in*_ is the sediment load in the inlet cross-section, 10^8^ t; *W*_*s, out*_ is the sediment load in the outlet cross-section, 10^8^ t; *W*_*s, d*_ is the sediment diversion, 10^8^ t. The data on sediment diversion are taken from the “*Hydrological Data of the Yellow River Basin*”^54^. This paper examines the addition of water from the Yiluo River, Qin River, and Dongping Lake to the LYR.

### Calculation of sediment delivery ratio

The sediment delivery ratio refers to the ratio of the amount of sediment output from a river reach to the total amount of sediment entering the river section during a specific period of time^58^. This paper utilizes the sediment delivery ratio to describe the sediment transport characteristics of the river channel, reflecting the overall indicators of sediment transport capacity, as well as the sedimentation characteristics of the river reach^6,44^. The sediment delivery ratio is mainly calculated based on 159 flood events, detailed as follows:

2 $$SDR={{{W_{s,out}}} \mathord{\left/ {\vphantom {{{W_{s,out}}} {{W_{s,in}}}}} \right. \kern-0pt} {{W_{s,in}}}}$$

When *SDR* > 1.0, the river reach is predominantly characterized by erosion; when *SDR* < 1.0, the river reach is predominantly characterized by aggradation; when *SDR* = 1.0, the river reach attains a dynamic equilibrium between erosion and aggradation. The higher the sediment delivery ratio, the more efficient the sediment transport in the river.

Processes such as sediment diversion or addition in the LYR often result in the sediment delivery ratio calculated from measured sediment volumes at the inlet and outlet cross-sections being the apparent sediment delivery ratio rather than the actual sediment delivery ratio. Therefore, this paper will use the following equation to calculate the actual sediment delivery ratio of the LYR:

3 $$SD{R_a}=\frac{{{W_{s,out}}+{W_{s,d}}}}{{{W_{s,in}}}}$$

where *SDR*_*a*_ represents the actual sediment delivery ratio. Applying Eq. (3) to recover the apparent sediment delivery ratio.

### Calculation of the incoming sediment coefficient

The incoming sediment coefficient *ξ* is defined as the ratio of sediment concentration to flow discharge, primarily illustrating the relationship between the river’s sediment transport capacity and fluctuations in water and sediment^20^.

4 $$\xi ={{{S_{in}}} \mathord{\left/ {\vphantom {{{S_{in}}} {{Q_{in}}}}} \right. \kern-0pt} {{Q_{in}}}}$$

where *S*_*in*_ is the inlet sediment concentration, kg/m^3^; *Q*_*in*_ is the import flow discharge, m^3^/s. The sediment concentration per unit flow discharge is greater if the incoming sediment coefficient is larger. This may result in the river channel becoming supersaturated, leading to aggradation. Conversely, it may exist in a sub−saturated state, resulting in scouring.

### Calculation of water load variation coefficient

The water load variation coefficient *η* is defined as the ratio of the outlet water load to the inlet water load:

5 $$\eta ={{{W_{w,out}}} \mathord{\left/ {\vphantom {{{W_{w,out}}} {{W_{w,in}}}}} \right. \kern-0pt} {{W_{w,in}}}}$$

where *W*_*w, in*_ and *W*_*w, out*_ are the inlet water load and outlet water load, respectively, 10^8^ m^3^. If the water load variation coefficient is greater than 1.0, it signifies a substantial increase in water load along the river reach; conversely, if the water load variation coefficient is less than 1.0, it means a considerable decrease in water load along the river reach.

### Calculation of cross-sectional morphology

**Fig. 2:**
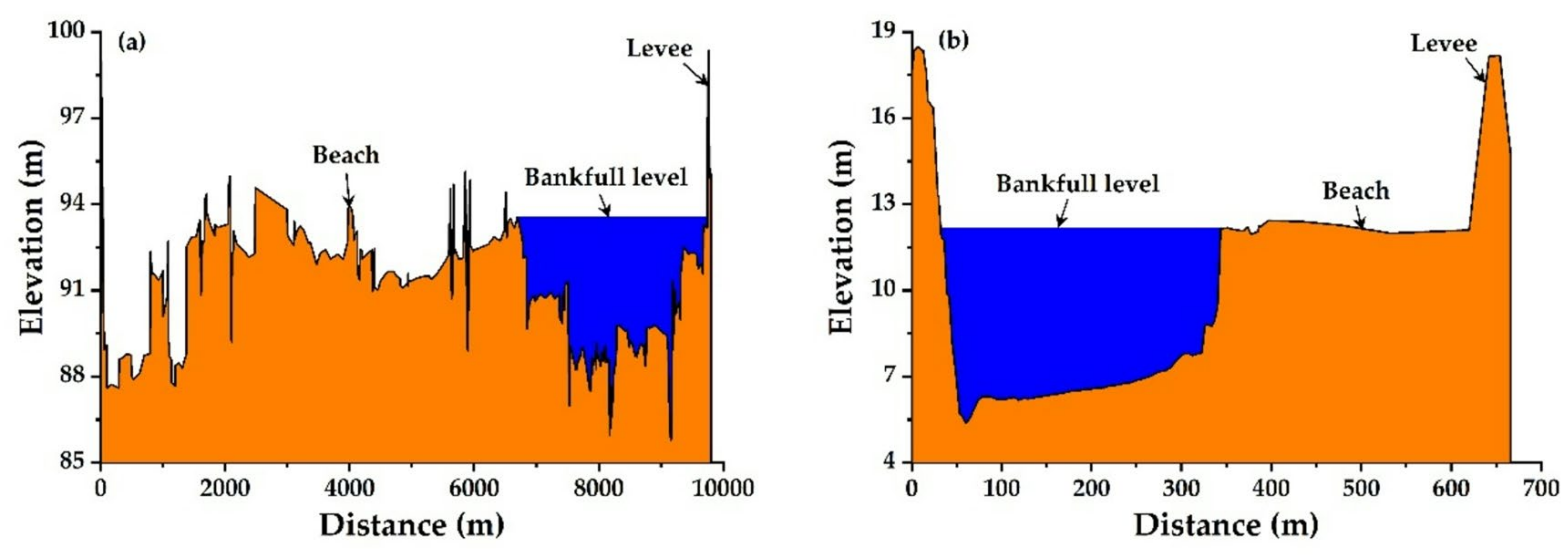
Cross-sectional shape in 2023. **(a)** Huayuankou, **(b)** Lijin.

The bankfull water level indicates the crucial point in a river where the water level reaches the threshold for overflowing from the trough. In this paper, the bankfull water level is determined based on the position and elevation of the flood plain lips on both banks. This paper defines the bankfull water level based on the position and elevation of the flood plain lips on both sides. It then calculates the indicators of the cross-sectional shape corresponding to the bankfull water level, including river width (*W*), depth (*D*), and the width-to-depth ratio (*δ* = *W*/*D*), as illustrated in Fig. 2. Given the differences in geometric morphology of different sections within the river section, this study adopts the reach−scale channel geomorphic parameters to reflect the general adjustment mode of the river reach. In reference to Xia et al.^61^, a reach−averaged method can be adopted in the calculation of cross-sectional parameters as follows:

6 $${G_{bf}}=exp\left[ {\frac{1}{{2L}}\sum\limits_{{i=1}}^{{N-1}} {\left( {lnG_{{bf}}^{i}+lnG_{{bf}}^{{i+1}}} \right)\vartriangle {x_i}} } \right]$$

where *G*_*bf*_ represents the average value of the cross-sectional shape indicators; *G*_*i, bf*_ and *G*_*i+1,bf*_ denote the actual morphological indicators corresponding to cross-sections *i* and *i* + 1, respectively. *Δx*_*i*_ signifies the distance between adjacent cross-sections (*i*, *i* + 1); *L* refers to the length of the river reach; *N* indicates the number of cross-sections. The morphological indicator in Eq. (6) refers to the river width *W*, water depth *D*, width-to-depth ratio *δ*, etc.

## Variations in water-sediment and riverbed boundary conditions of the LYR

### Variations in water-sediment conditions

**Fig. 3:**
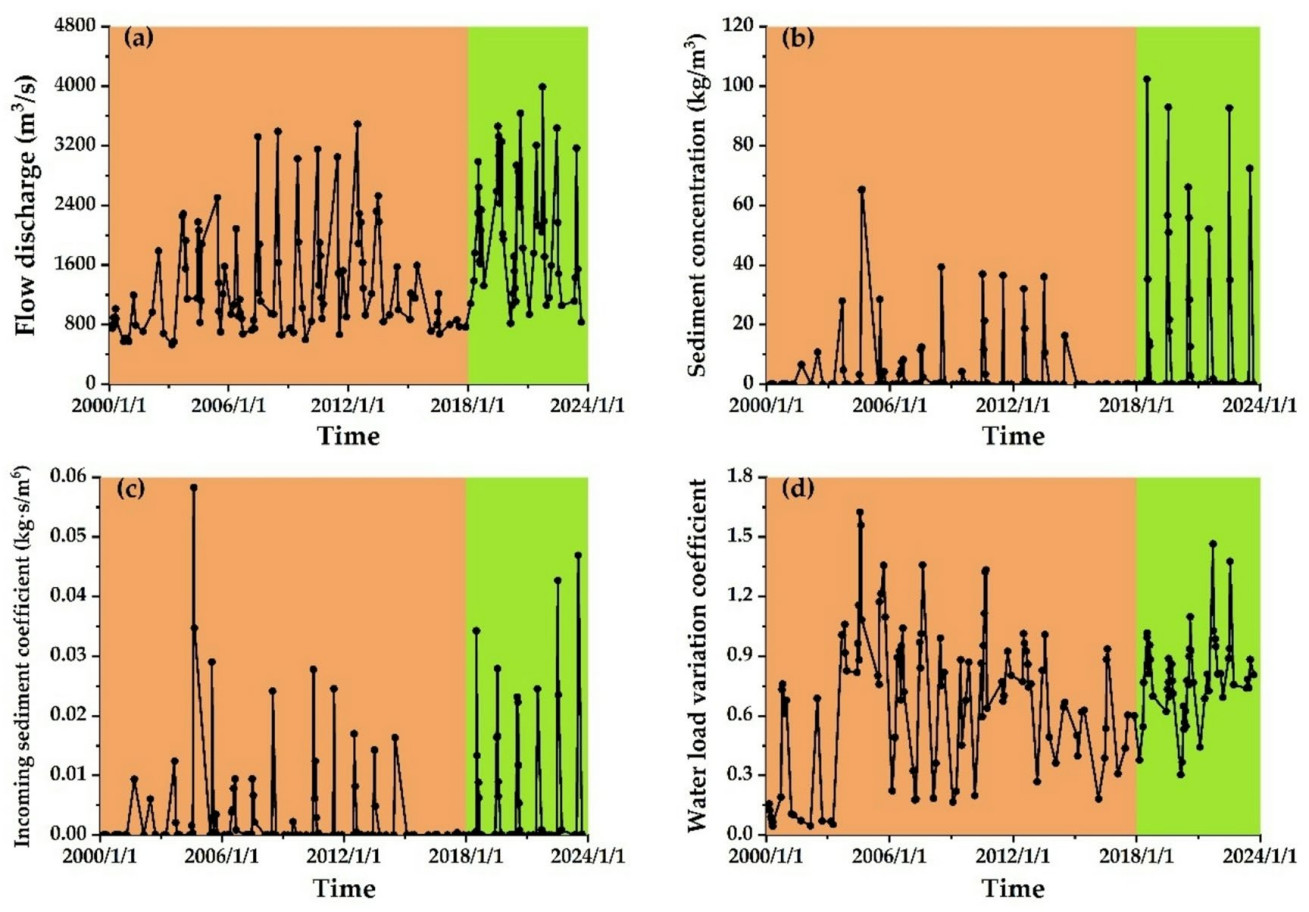
Water and sediment characteristics of flood events in the T − L reach. **(a)** Average flow discharge, **(b)** Average sediment concentration, **(c)** Incoming sediment coefficient, **(d)** Water load variation coefficient.

Figure 3 shows the runoff and sediment characteristics of flood events in the T − L reach. The water and sediment processes in the LYR are represented by the sum of three hydrological stations: Xiaolangdi Station on the mainstream, Heishiguan Station on the tributary of the Yiluo River, and Wuzhi Station on the tributary of the Qin River (referred to as Xiaoheiwu Station).

After 2000, the primary flow processes at Xiaoheiwu Station (including Xiaolangdi, Heishiguan, and Wuzhi Stations) predominantly occurred during the runoff and sediment regulation period (June to August). The floods primarily consisted of non−floodplain floods, with an average flow discharge range of 530 to 3992 m³/s, as illustrated in Fig. 3a. The average sediment concentration of flood events at Xiaoheiwu Station ranges from 0.0 to 102.4 kg/m³, with hyper−concentration mainly occurring during the pre−flood runoff and sediment regulation, as shown in Fig. 3b. Due to the implementation of the “multi−year sediment regulation and runoff and sediment management” method in Xiaolangdi Reservoir, floods characterized by high flow and sediment concentration occurred in 2000− 2014 and 2018 − 2023. From 2000 to 2014, the average flow discharge and average sediment concentration initially increased and then decreased. However, from 2018 to 2023, the concentrated sediment discharge from the Xiaolangdi Reservoir led to significant increases in the average flow discharge and sediment concentration.

Since 2000, the range of variation for the incoming sediment coefficient during flood events at Xiaoheiwu Station has been between 0.0 and 0.058 kg∙s/m^6^, primarily observed from 2000 to 2014 and from 2018 to 2023, as illustrated in Fig. 3c. Since 2000, the coefficient of runoff variation in the T − L reach has ranged from 0.044 to 1.625, showing an overall pattern of rising initially, then falling, and rising again. The water load variation coefficient was at its lowest in 2000, directly linked to flow interruptions in the LYR. During the period from 2018 to 2023, the overall trend of the water load variation coefficient showed an increasing trend, indicating that, in recent years, the water intake in the LYR has gradually increased, as shown in Fig. 3d.

### Variations in riverbed boundary conditions

**Fig. 4:**
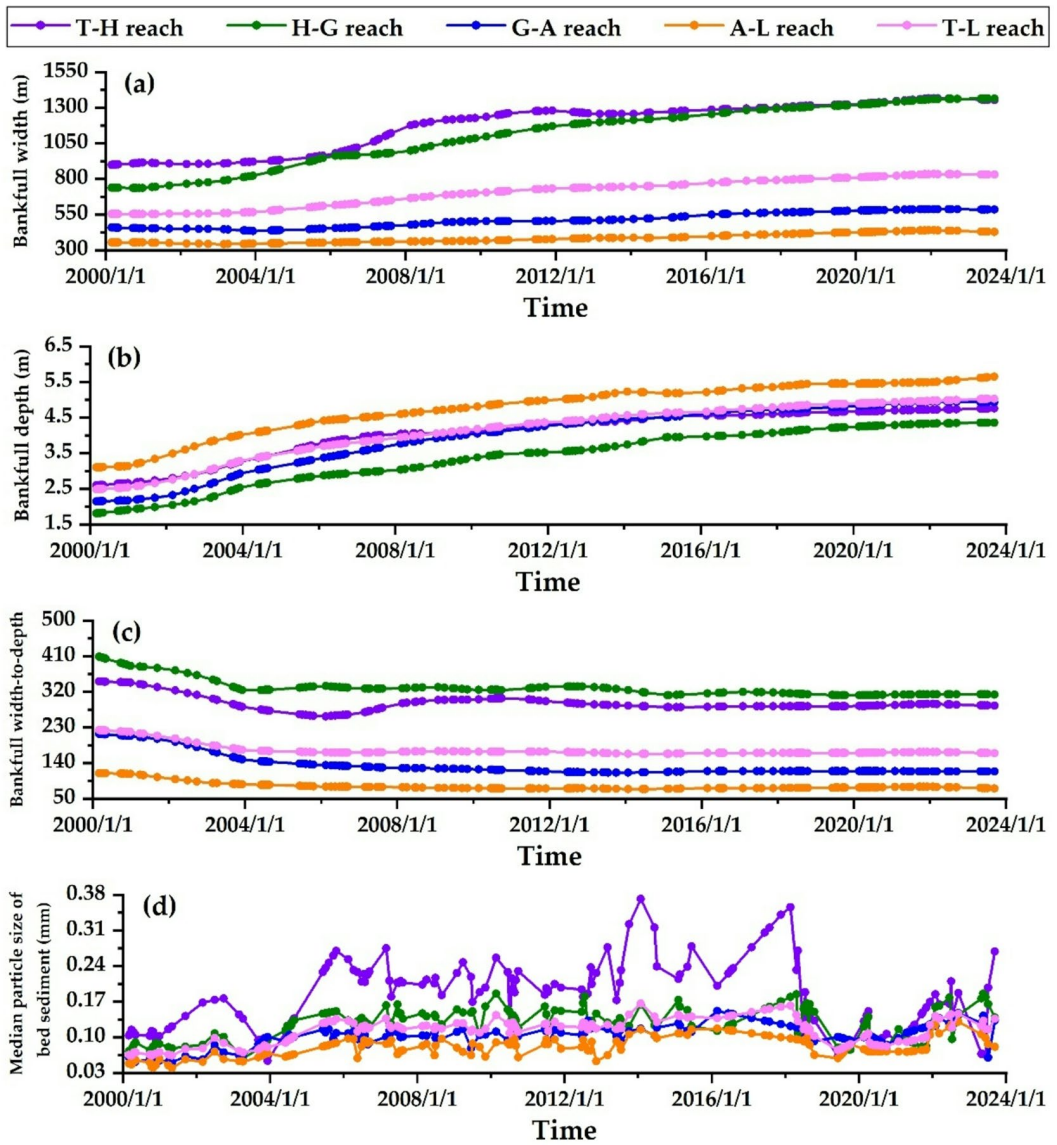
Temporal−spatial variations of cross-sectional morphology and median particle size of bed sediment in each reach. **(a)** Bankfull width, **(b)** Bankfull depth, **(c)** Bankfull width-to-depth ratio, **(d)** Median particle size of bed sediment.

Figure 4 shows temporal−spatial variations of cross-sectional morphology and median particle size of bed sediment in each reach. Since the closure of Xiaolangdi Reservoir in 1999, its primary focus has been on runoff storage and sediment retention. About 70% of fine sediment and more than 95% of medium coarse sediment are retained in the reservoir, significantly reducing sediment flow downstream. The river channel downstream has faced continuous erosion, gradually increasing the bankfull width and depth. In the T − L reach, the bankfull width is largest in the T − H reach and H − G reach (with a width of 1360 m), while the bankfull width is the narrowest in the A − L reach (with a width of 428 m), as shown in Fig. 4a. In the T − L reach, the bankfull depth in the A − L reach is the highest at 5.65 m, while the bankfull depth in the H − G reach is the lowest at 4.36 m, as shown in Fig. 4b. Since the Xiaolangdi Reservoir began operation, the bankfull width has increased by 1.5 times, and the depth has doubled, indicating that the river channel is generally developing in a “narrow and deep” direction.

The bankfull width-to-depth ratio is a crucial parameter that reflects river morphology, indicating whether the channel is “wide and shallow” or “narrow and deep”. Due to factors such as water-sediment conditions and reservoir operations, the bankfull width-to-depth ratio in the H − G reach is relatively large (with a width-to-depth ratio of 313.35). In contrast, the bankfull width-to-depth ratio of the A − L reach is relatively small (with a width-to-depth ratio of 75.86). From 2000 to 2006, the bankfull width-to-depth ratio in each river reach significantly decreased, indicating that the intensity of vertical scouring was greater than that of horizontal widening. From 2006 to 2023, the change in the bankfull width-to-depth ratio in each river reach was relatively minor, indicating that the width and depth of the river increased simultaneously during this period. Following adjustments to the bankfull depth and width, the primary channels in various reaches of the LYR have been deepened and laterally expanded since 2000. However, depth cutting has primarily occurred^62^, as illustrated in Fig. 4c. After 2000, severe erosion often leads to significant coarsening of downstream riverbeds, and the coarsening process varies depending on the original riverbed’s composition. During the scouring process, fine sediment particles are eliminated, while coarse sediment particles slowly accumulate on the bed surface, forming a coarsening layer that resists scouring^20^. The operation of the Xiaolangdi Reservoir has resulted in different levels of bed sediment coarsening in the LYR. The closer one is to the reservoir, the greater the degree of coarsening of the bed sediment. In the T − H reach, the median particle size of bed sediment exhibits the most significant coarsening, increasing from 0.105 mm in 2000 to 0.356 mm in 2018, indicating a particle size increase of approximately 3.5 times. Since 2018, the continuous sediment discharge from the Xiaolangdi Reservoir has significantly impacted bed sediment, resulting in a decrease in the median particle size to 0.079 mm. The increase in the median particle size of bed sediment in the H − G, G − A reaches, and A − L reach is relatively small, ranging from 1 to 1.5 times, as illustrated in Fig. 4d.

**Fig. 5:**
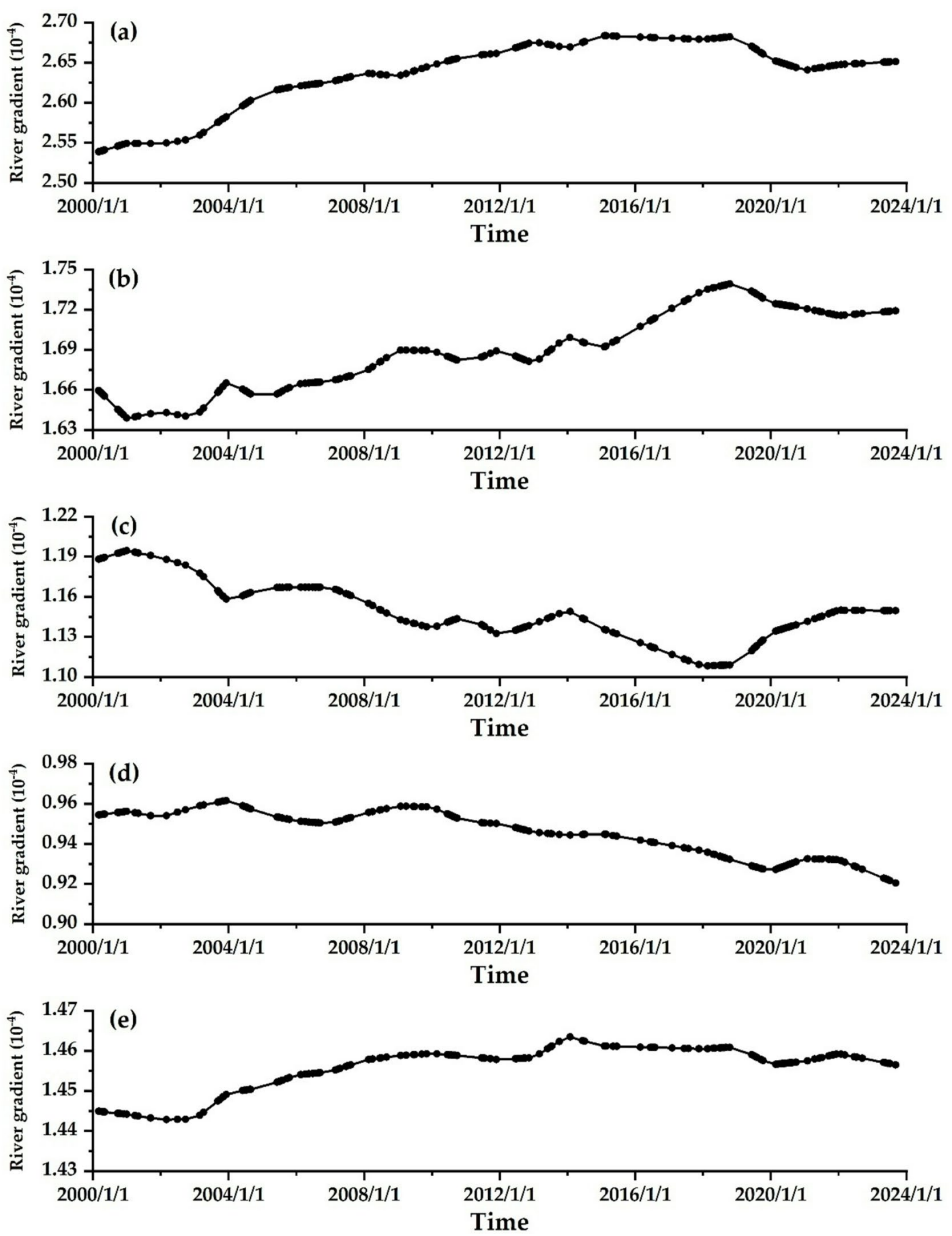
Spatial−temporal variations of river gradients in various river reaches. **(a)** T − H reach, **(b)** H − G reach, **(c)** G − A reach, **(d)** A − L reach, **(e)** T − L reach.

Figure 5shows the spatial−temporal variations of river gradients in various river reaches. River gradient refers to the drop value within a unit length of a river and is a core parameter that describes the characteristics of the longitudinal slope of a river^2,15^. Terrain conditions mainly affect the gradient of alluvial rivers. Aggradation will gradually lessen the gradient, while erosion will progressively steepen it. While the gradient of a river significantly affects flood discharge and sediment transport capacity, short−term changes in the LYR’s river gradient are minimal. The gradient of the T − H reach shows a trend of first increasing and then decreasing. From 2000 to 2018, the reservoir was mainly used for “runoff storage and sediment retention,” while the river channel continued to erode, gradually raising the gradient. However, from 2018 to 2023, the continuous sediment discharge from the reservoir led to a slight decline in the gradient, as illustrated in Fig. 5a. The adjustment range of the river gradient in the H − G reach has decreased slightly and exhibits a relative lag, as described in Fig. 5b. The G − A reach and A − L reach show a decreasing trend in river gradient related to aggradation during the non−flood season, as illustrated in Fig. 5c and d. In the T − L reach, the river’s overall gradient increases, as shown in Fig. 5e.

## Relationship between sediment delivery ratio and water-sediment, riverbed boundary conditions

### Temporal−spatial variations in sediment delivery ratio during flood events

**Fig. 6:**
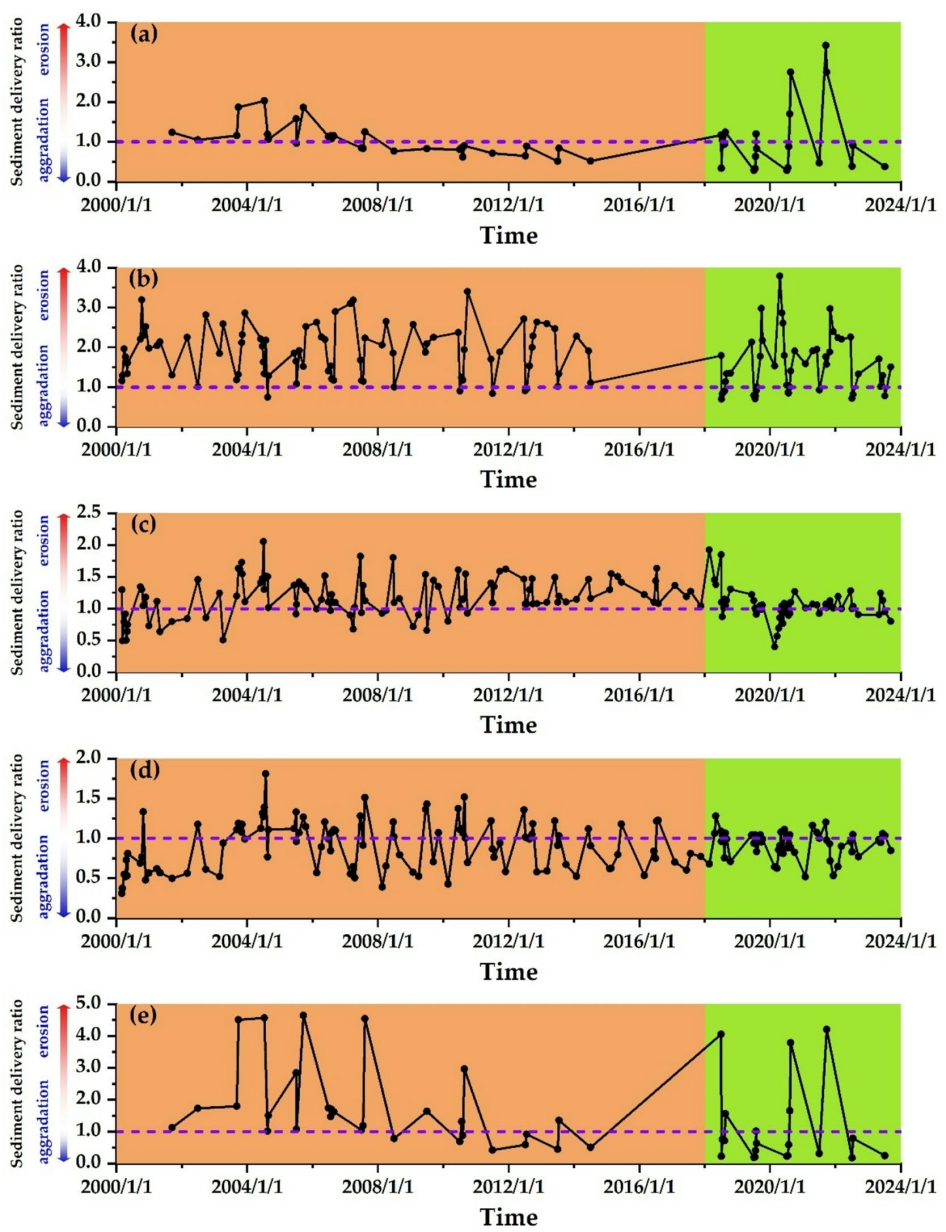
Spatial−temporal variations of sediment delivery ratios in each river reach. **(a)** T − H reach, **(b)** H − G reach, **(c)** G − A reach, **(d)** A − L reach, **(e)** T − L reach.

The variation of the sediment delivery ratio in the LYR is affected by water and sediment conditions. Due to the considerable interannual variation in water and sediment in the LYR, significant differences exist in the spatial and temporal distribution of the sediment delivery ratio. Equation (3) is used to calculate the sediment delivery ratio for different reaches of the LYR from 2000 to 2023, as shown in Fig. 6.

In the T − H reach, due to the water-sediment regulation of the Xiaolangdi Reservoir, the median particle size of bed sediment gradually increased between 2000 and 2014, reducing the erodibility of the riverbed and decreasing the supply of fine sediment to the runoff, resulting in a downward trend in the sediment delivery ratio over time. From 2018 to 2023, the ongoing sediment discharge from Xiaolangdi Reservoir increased the supply of fine sediment in the river channel, leading to a sharp rise in the sediment delivery ratio in the T − H reach, which exhibited significant fluctuations, as illustrated in Fig. 6a. In the H − G reach, the sediment delivery ratio ranges from 0.667 to 2.97, indicating substantial fluctuations between sedimentation in the river channel, as illustrated in Fig. 6b. In the G − A and A − L reaches, there was a notable fluctuation in the sediment delivery ratio from 2000 to 2022. However, from 2022 to 2024, the sediment delivery ratio remained approximately at 1.0, indicating that these river reaches have mainly achieved a balance between sedimentation, as illustrated in Fig. 6c and d. The sediment delivery ratio of the T − L reach aligns with the trend observed in the T − H reach. This indicates that the sedimentation processes in the T − H reach significantly impact the entire downstream river reach, as illustrated in Fig. 6e.

### Correlation analysis of sediment delivery ratio with water-sediment and riverbed boundary conditions

The Pearson correlation coefficient is commonly used to describe the relationship between two variables. The closer the absolute value of the correlation coefficient is to 1.0, the stronger the correlation^63^.

Factors affecting the sediment delivery ratio include water-sediment and riverbed boundary conditions. Water-sediment conditions encompass the incoming sediment coefficient (*ζ*) and the water load variation coefficient (*η*). In contrast, riverbed boundaries encompass the median particle size of bed sediment (*D*_*50*_), the width-to-depth ratio (*δ*), and the river channel gradient (*J*). Conduct a correlation analysis of the sediment delivery ratio of the river reach and its influencing factors. The study indicates that variables, including the incoming sediment coefficient, the median particle size of bed sediment, and the width-to-depth ratio, correlate significantly with the sediment delivery ratio in a negative manner. In contrast, factors such as the water load variation coefficient and river gradient exhibit a significant positive correlation with the sediment delivery ratio, as illustrated in Table 1.

**Table 1:** Pearson coefficient between sediment delivery ratio, water-sediment, and riverbed boundary.

Factor	T − H reach	H − G reach	G − A reach	A − L reach	T − L reach
Incoming sediment coefficient	−0.434**	−0.591**	−0.638**	−0.543**	−0.397**
Water load variation coefficient	0.074	0.344**	0.612**	0.823**	0.284**
Median particle size of bed sediment	−0.293**	−0.501**	−0.543**	−0.415**	−0.189**
Width-to-depth ratio	−0.409**	−0.708**	−0.588**	−0.395**	−0.288**
River gradient	0.144**	0.113**	0.617**	0.193**	−0.011**

Note: * indicates a significant correlation at the 0.05 level (bilateral), ** indicates a significant correlation at the 0.01 level (bilateral).

An increase in the median particle size of bed sediment enhances the riverbed’s resistance to erosion. It reduces the river’s ability to transport sediment, resulting in a negative correlation between the sediment delivery ratio and the median particle size of bed sediment. Since 2000, the median particle size of bed sediment in downstream rivers has increased significantly^64^, resulting in a notable rise in the impact of median particle size on the river’s sediment transport capacity. The sediment transport capacity increases along the river due to the narrowing of the river width and the rising flow velocity. At a specific flow discharge, the sediment transport capacity of a “wide shallow” reach is less than that of a “narrow deep” reach, indicating a negative correlation between the width-to-depth ratio and the sediment delivery ratio. The A − L reach features a “narrow deep” river channel, which weakens the correlation between the width-to-depth ratio and the sediment delivery ratio. Based on Manning’s equation, flow velocity and sediment transport capacity rise as the river gradient increases. Thus, a significant positive correlation exists between the river gradient and the sediment delivery ratio. The sediment delivery ratio in the G − A reach exhibits the strongest correlation with the river gradient among all other river reaches, with a correlation coefficient of 0.617. The correlation coefficient between river gradient and sediment delivery ratio in the T − L reach is relatively low, indicating that the variation of river gradient in this reach is relatively slight.

### Theoretical equation of the sediment delivery ratio

This paper develops the fundamental structural form of the theoretical expression for the sediment delivery ratio in river reaches, based on the sediment transport rates. Then, the key parameters are calibrated with measured data, resulting in a theoretical expression of the sediment delivery ratio for different downstream river reaches. During erosion and aggradation processes, the cross-sectional morphology, roughness, and material composition of the riverbed undergo certain modifications, which influence the sediment load that the runoff can transport. The sediment transported at the same flow discharge can vary several times to tens of times, depending on the incoming sediment load. Qian et al. ^**12**^ argued that the sediment transport pattern in the LYR differs from that of rivers with lower sediment concentrations. The sediment transport rate at the outlet cross-section is affected by the flow discharge at that reach and the sediment concentration at the upstream inlet cross-section:

7 $${Q_{s,out}}=KQ_{{out}}^{\alpha }S_{{in}}^{\beta }$$

where *Q*_*s, out*_ is the sediment transport rate, t/s; *Q*_*out*_ represents the flow discharge at the outlet cross-section, m^3^/s; *S*_*in*_ is the sediment concentration at the inlet cross-section, kg/m^3^; *K*, *α*, and *β* are coefficients.

The sediment load at the outlet cross-section of the reach, *W*_*s, out*_ (in units of 10⁸ t), can be expressed as:

8 $${W_{s,out}}=\frac{{24 \times 3600 \times T{Q_{s,out}}}}{{1{0^8} \times 1{0^3}}}=\frac{{86.4T{Q_{s,out}}}}{{1{0^8}}}$$

where *T* is the flood duration, d.

Substituting Eq. (7) into Eq. (8) yields:

9 $${W_{s,out}}=\frac{{24 \times 3600 \times TKQ_{{out}}^{\alpha }S_{{in}}^{\beta }}}{{1{0^8} \times 1{0^3}}}=\frac{{86.4TKQ_{{out}}^{\alpha }S_{{in}}^{\beta }}}{{1{0^8}}}$$

The sediment load at the inlet cross-section of the reach, *W*_*s, in*_ (in units of 10⁸ t), can be expressed as:

10 $${W_{s,in}}=\frac{{24 \times 3600 \times T{Q_{s,in}}}}{{1{0^8} \times 1{0^3}}}=\frac{{86.4T{Q_{s,in}}}}{{1{0^8}}}$$

where *Q*_*s, in*_ is the sediment transport rate at the inlet cross-section of the reach, t/s.

Substituting Eq. (9) and Eq. (10) into Eq. (2) produces the theoretical expression for the sediment delivery ratio of the reach.

11 $$SDR=\frac{{{W_{s,out}}}}{{{W_{s,in}}}}=\frac{{KQ_{{out}}^{\alpha }S_{{in}}^{\beta }}}{{{Q_{s,in}}}}=\frac{{KQ_{{out}}^{\alpha }S_{{in}}^{\beta }}}{{{Q_{in}}{S_{in}}}}=K{\left( {\frac{{{S_{in}}}}{{{Q_{in}}}}} \right)^{\beta -1}}{\left( {\frac{{{Q_{out}}}}{{{Q_{in}}}}} \right)^\alpha }Q_{{in}}^{{\alpha +\beta -2}}$$

where: *Q*_*in*_ is the flow discharge at the inlet cross-section of the reach, m³/s.

The water load at the inlet cross-section of the reach, *W*_*w, in*_ (in units of 10⁸ m^3^), can be expressed as:

12 $${W_{w,in}}=\frac{{24 \times 3600 \times T{Q_{in}}}}{{1{0^8}}}=\frac{{86400T{Q_{in}}}}{{1{0^8}}}$$

The water load at the outlet cross-section of the reach, *W*_*w, out*_ (in units of 10⁸ m^3^), can be expressed as:

13 $${W_{w,out}}=\frac{{24 \times 3600 \times T{Q_{out}}}}{{1{0^8}}}=\frac{{86400T{Q_{out}}}}{{1{0^8}}}$$

From Eqs. (12) and (13), we derive the following:

14 $$\frac{{{W_{w,out}}}}{{{W_{w,in}}}}=\frac{{{Q_{out}}}}{{{Q_{in}}}}$$

Substituting Eq. (14) into Eq. (11) yields:

15 $$SDR=\frac{{{W_{s,out}}}}{{{W_{s,in}}}}=\frac{{KQ_{{out}}^{\alpha }S_{{in}}^{\beta }}}{{{Q_{in}}{S_{in}}}}=K{\left( {\frac{{{S_{in}}}}{{{Q_{in}}}}} \right)^{\beta -1}}{\left( {\frac{{{W_{w,out}}}}{{{W_{w,in}}}}} \right)^a}Q_{{in}}^{{\alpha +\beta -2}}$$

By substituting Eqs. (4) and (5) into Eq. (16), we can obtain:

16 $$SDR=\frac{{KQ_{{out}}^{\alpha }S_{{in}}^{\beta }}}{{{Q_{in}}{S_{in}}}}=K{\xi ^{\beta -1}}{\eta ^\alpha }Q_{{in}}^{{\alpha +\beta -2}}$$

From Eq. (16), it is evident that the sediment delivery ratio is dependent on *ξ*, *η*, and *Q*_*in*_. Using flood sample data from 2000 to 2023, the sediment delivery ratio is analyzed, and the equation structure (15) is tested. Xiaoheiwu Station (Xiaolangdi Station, Heishiguan Station, and Wuzhi Station) is considered the inlet cross-section, while Lijin Station is recognized as the outlet cross-section.

From 2000 to 2023, there were 159 flood events in the LYR, with an average flow range of 530 to 3992 m³/s and an average sediment concentration range of 0.0 to 102.4 kg/m³. According to existing research^59^, clear water is released from the river channel when the average sediment concentration during a flood event is below 1.0 kg/m³. The average sediment concentration of the selected flood events used to construct the theoretical relationship of sediment delivery ratio in this paper is greater than 1.0 kg/m^3^. Statistics show there were 49, 131, 159, and 159 floods with an average sediment concentration exceeding 1.0 kg/m³ in the T − H, H − G, G − A, and A − L reaches, respectively.

In the T − L reach, there are 49 flood events with an average sediment concentration exceeding 1.0 kg/m³, with an average flow rate ranging from 708 to 3992 m³/s and an average sediment concentration between 1.46 and 102.40 kg/m³. A nonlinear multiple regression analysis was conducted using the measured runoff and sediment data to establish the theoretical equation for the sediment delivery ratio of the T − L reach. The main coefficients of the equation are provided in Table 2.

**Table 2:** Regression parameters of the sediment delivery ratio equation.

Parameters		K	β − 1	α	α + β − 2
Condition 1	Regression value	0.084	−0.773	1.761	0.016
	P−value	7.26 × 10^− 8^	1.54 × 10^− 14^	5.38 × 10^− 45^	0.856
Condition 2	Regression value	0.072	−0.624	1.647	
	P−value	7.06 × 10^− 7^	5.19 × 10^− 16^	3.54 × 10^− 55^	

The test of the regression equation coefficients indicates that *K*, *β* − 1, and α all pass the hypothesis test, with the P-value of the t-test not exceeding 10^− 5^, which suggests that *K*, *β* − 1, and α cannot be 0. However, the t−statistic of *α* + *β* − 2 is not significant, and the P−value of the t−test is 0.856, indicating that the *Q*_*in*_ term in Eq. (16) may be removed. In summary, based on the average scale of the flood events, Eq. (16) was fitted to the T-L reach, where *α* + *β* = 2 also has a statistical basis.

For the parameters *α* and *β*, Qian et al.^12^ suggested that during the discharge of clear runoff from the Sanmenxia Reservoir in the LYR during the non-flood season, the sediment transport rate is directly proportional to the square of the flow discharge; that is, *β* = 0 and *α*= 2.- During flood events, the sediment transport rates at Gaocun and Sunkou Stations are α + β = 1.96 and 1.94, respectively. The relationship between the sediment transport rates at Gaocun Station and Lijin Station during the flood season, as obtained by Zhang et al.^65^, shows that *α* + *β* equals 1.96 and 2.08, respectively. Zhao et al.^66^ analyzed flood data from downstream rivers during the flood season and found that the three stations of Gaocun, Aishan, and Lijin had *α* + *β* values of 1.94, 2.03, and 2.05, respectively. Leopold et al.^67^ and Knighton et al.^68^ also demonstrated that *α* + *β* fluctuated around 2.0, suggesting that the *Q*_*in*_ term in Eq. (16) may be eliminated.

Based on the analysis above, the theoretical equation for sediment delivery ratio in the T − L reach is as follows:

17 $$SDR=0.118{\xi ^{-0.513}}{\eta ^{1.525}},{R^2}=0.831$$

Based on Eq. (17), the fundamental structural form of the sediment delivery ratio can be derived as:

18 $$SDR=K{\xi ^{\beta -1}}{\eta ^\alpha }$$

From Eq. (18), the key factors influencing the sediment delivery ratio are the incoming sediment coefficient and the water load variation coefficient, which is consistent with the main conclusion of the chapter on the correlation analysis of sediment delivery ratio with water-sediment and riverbed boundary conditions.

Based on the measured runoff and sediment data from floods in the LYR between 2000 and 2023, theoretical equations were created to describe the relationship between the sediment delivery ratio and the runoff and sediment conditions in each river reach. The coefficients *K*, *α*, and *β* in Eq. (18) were fitted, as detailed in Table 3. According to Table 3, the fitting results from this study indicate that *α* + *β* equals 2.0, further confirming that the parameters in this study are fundamentally correct.

**Table 3:** Fitting parameters of the theoretical equation for the sediment delivery ratio.

Reach	K	β	α	α + β	*R* ^2^
T − H reach	0.137	0.569	1.417	1.986	0.813
H − G reach	0.173	0.626	1.369	1.995	0.826
G − A reach	0.239	0.665	1.351	2.016	0.809
A − L reach	0.289	0.691	1.317	2.008	0.867
T − L reach	0.118	0.487	1.525	2.012	0.831

Numerous factors and complex mechanisms influence the sediment delivery ratio along the LYR^12,29,35,36,39,42–44,69^. The main viewpoints are as follows: (1) The conditions of incoming runoff and sediment are the main factors affecting the sediment delivery ratio. Qian et al.^12^ believed that the main influencing factors of sediment discharge are the flow discharge and the incoming sediment coefficient. Wu et al.^42^ considered the incoming sediment coefficient and water load variation coefficient to be the primary influencing factors. Fei et al.^43^ and Fu et al.^44^ thought that the incoming sediment coefficient is the most critical factor; (2) Under the given conditions of runoff and its processes, the characteristics that affect the sediment carrying capacity of runoff, such as flow velocity, flow shear force, and energy consumption, mainly depend on the riverbed boundary, such as cross-sectional shape, river gradient, and roughness^69^. The impact of the riverbed boundary on the sediment delivery ratio must not be overlooked.

River sediment transport is a complex phenomenon. The sediment delivery ratio is generally influenced by the water-sediment and riverbed boundaries, reflecting the river’s capacity to transport runoff and sediment under specific boundary conditions^44^. Existing research on the sediment delivery ratio has extensively examined the effects of incoming runoff and sediment conditions, but has given less attention to the combined impact of riverbed boundary conditions^47^. As a sediment−laden alluvial river, the LYR often exhibits a strong coupling effect between runoff and sediment movement, as well as riverbed deformation. This coupling effect becomes more pronounced, especially when a substantial change in the volume of upstream water and sediment results in severe deformation of the riverbed^70^.

According to existing research, the coefficients *K*, *α*, and *β* in Eq. (11) indirectly reflect the influence of riverbed boundary conditions. The coefficient *K* is primarily associated with the accumulation of sediment in the river channel. In contrast, the coefficients α and *β* are primarily associated with factors such as the shape of the river channel reach and the distance along the route^47^. The coefficients *K*, *α*, and *β* for various river reaches often vary significantly, and a certain level of uncertainty exists^70^. Based on existing research, this paper proposes an empirical equation for the sediment delivery ratio, which includes the median particle size of bed sediment, the width-to-depth ratio, and the river gradient:

19 $$SDR=a{\delta ^b}{\xi ^{\left( {c{D_{50}}+d} \right)}}{\eta ^{\left( {eJ+f} \right)}}$$

where *D*_*50*_ represents the median particle size of bed sediment, m; *δ* indicates the width-to-depth ratio; *J* denotes the river gradient; *a*, *b*, *c*, *d*, *e*, and *f* are all coefficients.

The theoretical expression for the sediment delivery ratio of river reaches developed in this study emphasizes the importance of channel boundaries. However, parameters such as the median particle size of bed sediment, the width-to-depth ratio, and the river gradients vary significantly along the course of the LYR^34,35^. Considering the entire lower reach as possessing uniform channel boundaries would inadequately capture its inherent characteristics. Therefore, based on the riverbed evolution characteristics of various reaches, this study formulates distinct theoretical expressions for the sediment delivery ratio corresponding to specific reaches of the LYR (T − H, H − G, G − A, and A − L). This paper employs nonlinear regression to fit theoretical equations of the sediment delivery ratio and the relationships among water-sediment and riverbed boundaries, based on measured data from flood events between 2000 and 2023 across various river reaches. The specific fitting parameters are detailed in Table 4.

**Table 4:** Fitting parameters of the theoretical equation for the sediment delivery ratio.

Reach	a	b	c	d	e	f	*R* ^2^
T − H reach	32.462	−0.925	217.748	−0.453	1144.458	1.379	0.874
H − G reach	9.853	−0.692	97.104	−0.383	123.000	1.351	0.886
G − A reach	1.637	−0.390	53.901	−0.343	54.669	1.342	0.874
A − L reach	0.871	−0.262	6.988	−0.302	27.075	1.293	0.865

According to the table, this paper presents parameters such as the median particle size of bed sediment, the width-to-depth ratio, and the channel’s gradient. Considering the riverbed boundary enhances the correlation between calculated and measured values compared to not taking it into account. This indicates that channel sedimentation is closely linked to the water-sediment and the riverbed boundary. Due to the low median particle size of bed sediment and the river gradient, the combined indices of the incoming sediment coefficient and the water load variation coefficient equal 2.0. This indicates that the theoretical equation for the sediment delivery ratio is more valid after expanding the riverbed boundary.

## Discussions

### Applicability testing of the sediment delivery ratio

To further clarify the applicability of the theoretical equation for the sediment delivery ratio, considering the river boundaries constructed in this article, this article presents existing research findings on the theoretical equations for the sediment delivery ratio of flood events, specifically addressing downstream river channel erosion and aggradation.

Fu et al.‘s equation for calculating sediment delivery ratio^44^:

20 $$SDR=0.0818{\left( {\frac{{{S_{in}}}}{{{Q_{in}}}}} \right)^{-0.559}}$$

Zhang et al.‘s equation for calculating the sediment delivery ratio^36^:

21 $$SDR=0.1{\left( {\frac{{{Q_{in}}}}{{{S_{in}}}}} \right)^{0.56}}{\left( {\frac{{{W_{w,out}}}}{{{W_{w,in}}}}} \right)^{1.004}}$$

Li et al.‘s equation for calculating the sediment delivery ratio^39^:

22 $$SDR=\frac{{35Q_{{in}}^{{0.45}}{P^{0.4}}{{\left( {{{{Q_{out}}} \mathord{\left/ {\vphantom {{{Q_{out}}} {{Q_{in}}}}} \right. \kern-0pt} {{Q_{in}}}}} \right)}^{0.6}}}}{{S_{{in}}^{{0.64}}}}$$

where *P* is the content of fine sediment particles, %.

The existing research results and the theoretical equation of sediment delivery ratio constructed in this paper were used to calculate the erosion and aggradation of different flood events in the T − L reach after the operation of Xiaolangdi Reservoir.

The equation for calculating the amount of erosion and aggradation during a flood event is as follows^44^:

23 $$\varDelta {W_s}={W_{s,in}}\left( {1-SDR} \right)$$

This study applies the theoretical expressions of sediment delivery ratio to the four river reaches (T − H, H − G, G − A, and A − L) to estimate the total sedimentation load across the entire reach. The following formula gives the total erosion or aggradation, *ΔW*_*s*_, across the whole reach:

24 $$\varDelta {W_s}=\varDelta {W_s}_{1}+\varDelta {W_s}_{2}+\varDelta {W_s}_{3}+\varDelta {W_s}_{4}$$

where.

$$\varDelta {W_s}_{1}=\frac{{86.4T{S_{in1}}{Q_{in1}}\left( {1-SD{R_1}} \right)}}{{1{0^8}}}$$

$$\varDelta {W_s}_{2}=\frac{{86.4T{S_{in2}}{Q_{in2}}\left( {1-SD{R_2}} \right)}}{{1{0^8}}}$$

$$\varDelta {W_s}_{3}=\frac{{86.4T{S_{in3}}{Q_{in3}}\left( {1-SD{R_3}} \right)}}{{1{0^8}}}$$

$$\varDelta {W_s}_{4}=\frac{{86.4T{S_{in4}}{Q_{in4}}\left( {1-SD{R_4}} \right)}}{{1{0^8}}}$$

where *ΔW*_*s1*_, *ΔW*_*s2*_, *ΔW*_*s3*_, and *ΔW*_*s4*_ represent the sedimentation load of flood events for the four river reaches, respectively, 10^8^ t; *S*_*in1*_, *S*_*in2*_, *S*_*in3*_, and *S*_*in4*_ represent the average sediment concentrations at the inlet cross-sections of the four river reaches during flood events, respectively, kg/m³; *Q*_*in1*_, *Q*_*in2*_, *Q*_*in3*_, and *Q*_*in4*_ represent the average flow discharges at the inlet cross-sections of the four river reaches during flood events, respectively, m³/s; *SDR*_*1*_, *SDR*_*2*_, *SDR*_*3*_, and *SDR*_*4*_ represent the sediment delivery ratios of the four river reaches during flood events, respectively.

Considering that the four river reaches are contiguous, with the sediment load at the outlet of each upstream reach being equivalent to that at the inlet of the next downstream reach, Eq. (24) can be derived as follows:

25 $$\varDelta {W_s}=\frac{{86.4T\left( {{S_{in1}}{Q_{in1}}-{S_{in4}}{Q_{in4}}SD{R_4}} \right)}}{{1{0^8}}}$$

The average sediment concentration at the inlet of the Aishan to Lijin reach, *S*_*in4*_, can be expressed as:

26 $${S_{in4}}=\frac{{{S_{in1}}{Q_{in1}}SD{R_1}SD{R_2}SD{R_3}}}{{{Q_{in4}}}}$$

Substituting Eq. (26) into Eq. (25) yields:

27 $$\varDelta {W_s}=\frac{{86.4T{S_{in1}}{Q_{in1}}\left( {1-SD{R_1}SD{R_2}SD{R_3}SD{R_4}} \right)}}{{1{0^8}}}$$

Equation (27) is derived by simultaneously calculating the sediment delivery ratios for the four river reaches. It mainly aims to determine the total erosion or aggradation in the entire lower reach.

**Fig. 7:**
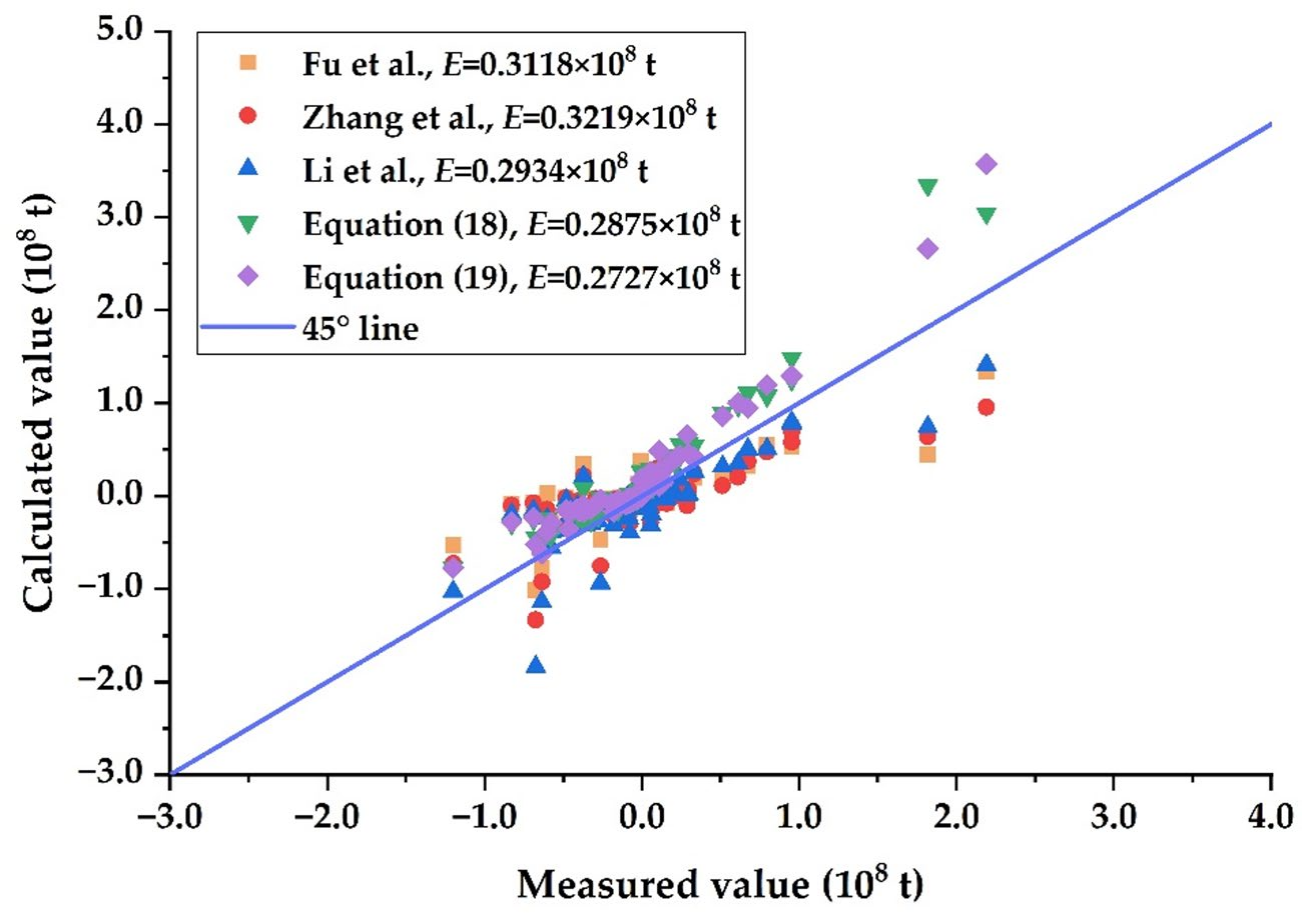
Verification of different sediment delivery ratio equations using flood data.

Figure 7 shows the results of the erosion and deposition calculated by the flood sediment delivery ratio from Equations (18) to (22), and also provides the average error *E* (i.e., the average distance from the 45° line) defined as follows^44^:

28 $$E=\sqrt {\frac{1}{N}\sum\limits_{{i=1}}^{N} {{{\left( {\vartriangle {W_{s,ic}}-\vartriangle {W_{s,im}}} \right)}^2}} }$$

where *∆W*_*s, ic*_ is calculated values of erosion and aggradation, 10^8^ t; *∆W*_*s, im*_ is measured values of erosion and aggradation, 10^8^ t. *N* is the number of flood events.

According to Fig. 7, the average errors *E* of Eqs. (19) and (18) are 0.2727 × 10^8^ t and 0.2875 × 10^8^ t, respectively, indicating that the calculated results of the two equations are in good agreement with the measured data. Compared with the average error *E* of Eq. (18), Fu et al.‘s Eq^44^., Zhang et al.‘s Eq^36^., and Li et al.‘s Eq^39^., the average error *E* of Eq. (19) is relatively small, indicating that the theoretical equation for sediment delivery ratio constructed in this paper has good applicability in solving the sediment delivery ratio in the LYR.

### Impact of incoming sediment coefficient on sediment delivery ratio

Under the current conditions, the riverbed boundary conditions for each reach of the LYR are shown in Table 5.

**Table 5:** Current riverbed boundary conditions of each river reach.

Reach	Median particle size of bed sediment (mm)	Bankfull width-to-depth	River gradient (10^− 4^)
T − H reach	0.168	344	2.63
H − G reach	0.117	338	1.69
G − A reach	0.099	143	1.15
A − L reach	0.079	60	0.95
T − L reach	0.107	191	1.45

**Fig. 8:**
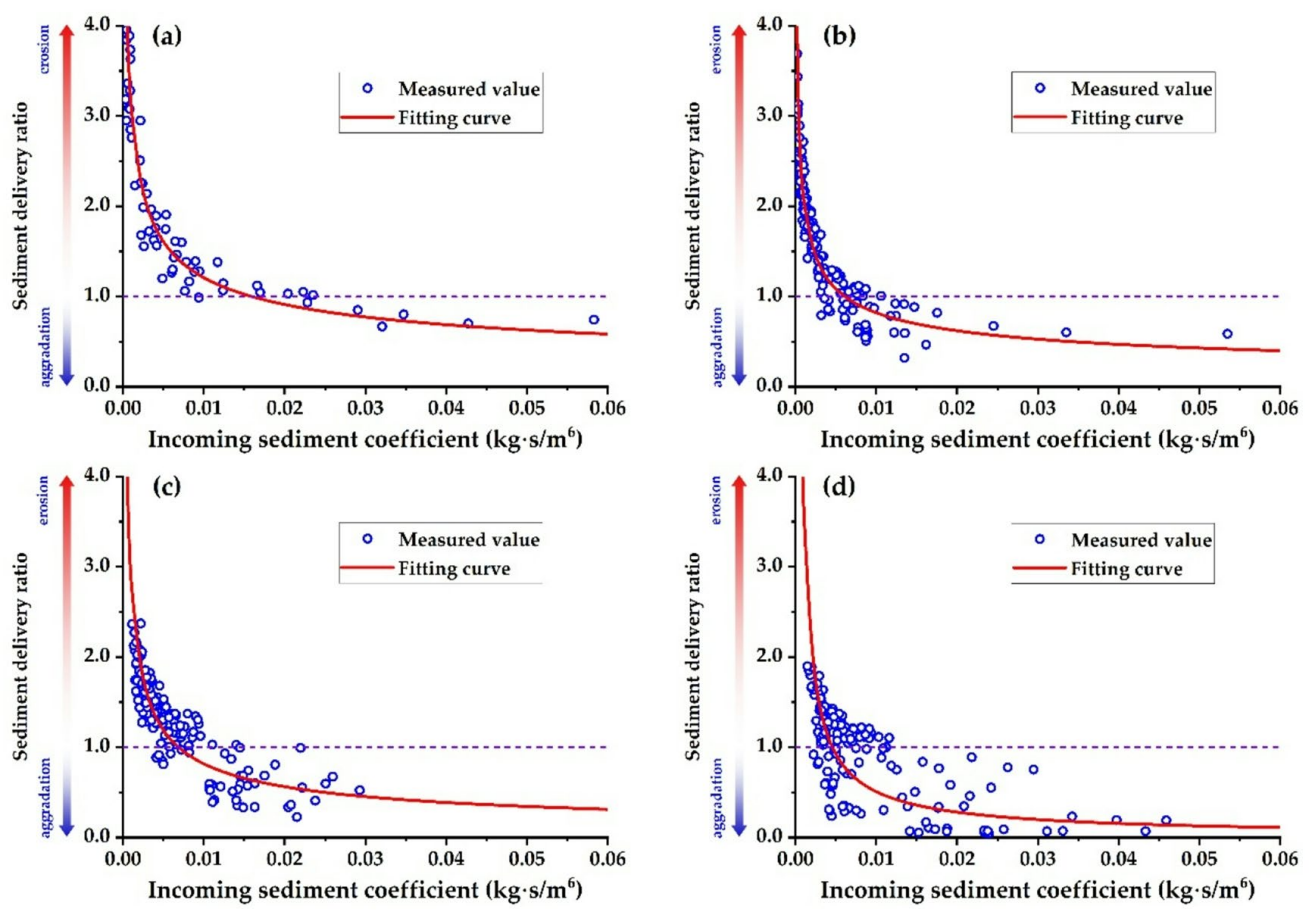
Relationship curve between sediment delivery ratio and incoming sediment coefficient. **(a)** T − H reach, **(b)** H − G reach, **(c)** G − A reach, **(d)** A − L reach.

Figure 8 illustrates the relationship between the sediment delivery ratio and the incoming sediment coefficient for each river reach in a scatter plot. The graph demonstrates that the sediment delivery ratio progressively diminishes as the incoming sediment coefficient increases, indicating an exponential correlation between the two variables.

**Fig. 9:**
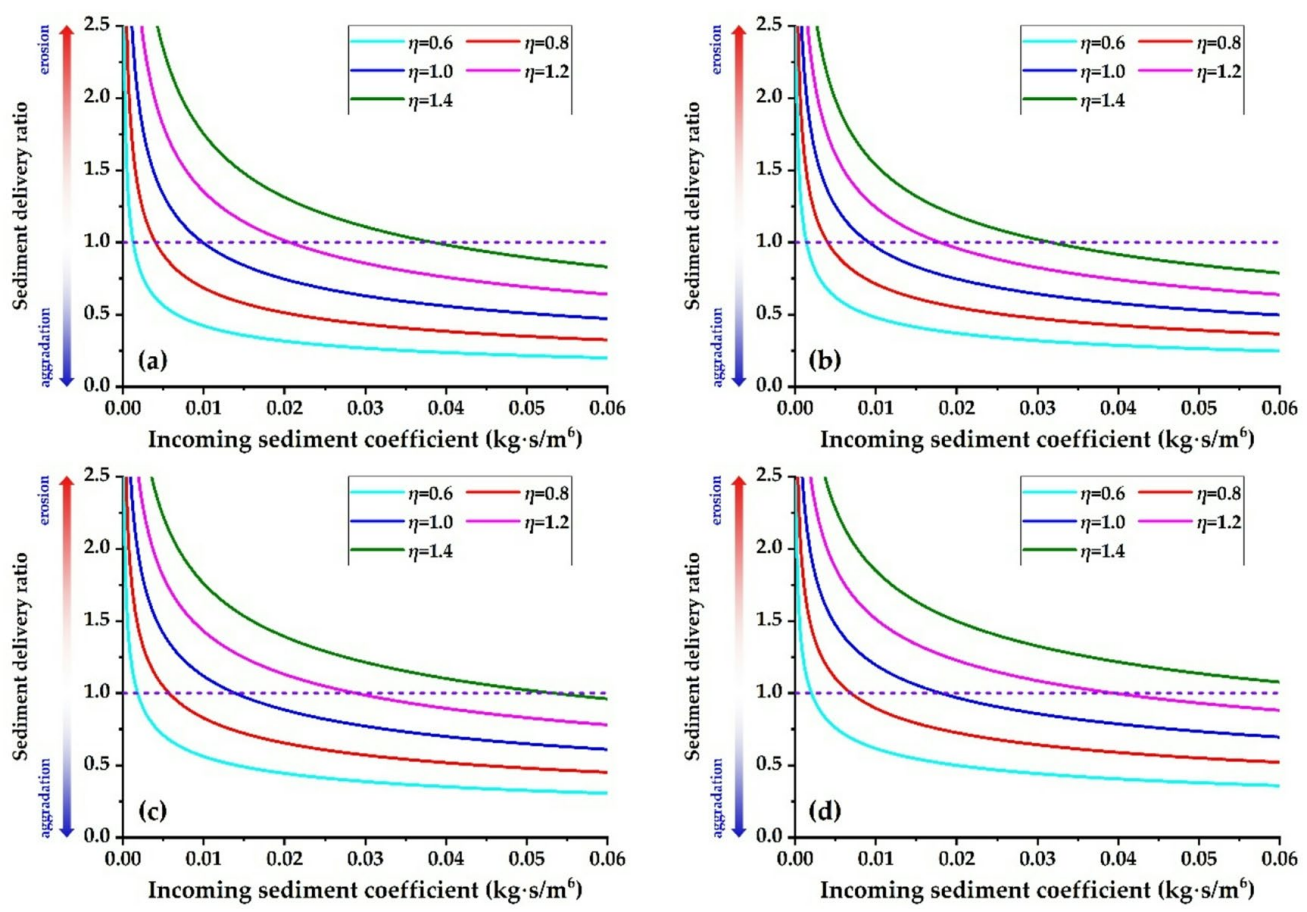
Relationship between incoming sediment coefficient and Sediment Delivery Ratio in the various reaches. **(a)** T − H reach, **(b)** H − G reach, **(c)** G − A reach, **(d)** A − L reach.

Figure 9 shows the relationship between the incoming sediment coefficient and the sediment delivery ratio. As the incoming sediment coefficient increases, the sediment delivery ratio diminishes exponentially. Under the same incoming sediment coefficient conditions, the sediment delivery ratio demonstrates a gradually increasing trend as the water load variation coefficient increases.

**Fig. 10:**
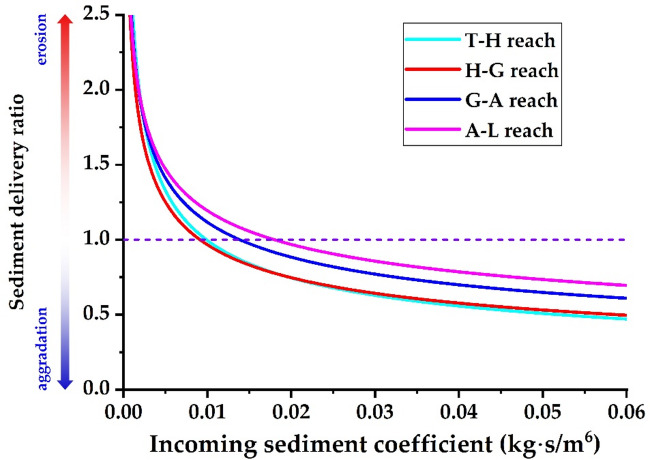
Relationship between the incoming sediment coefficient and the sediment delivery ratio (*η* = 1.0).

Figure 10 illustrates the relationship between the incoming sediment coefficient and the sediment delivery ratio across various river reaches when the water load variation coefficient is 1.0. Given current boundary conditions—including the width-to-depth ratio, median particle size of bed sediment, and river gradients—the critical incoming sediment coefficients that correspond to a sediment delivery ratio of 1.0 are 0.0098 kg∙s/m⁶ for the T − H reach, 0.0091 kg∙s/m⁶ for the H − G reach, 0.0138 kg∙s/m⁶ for the G − A reach, and 0.0180 kg∙s/m⁶ for the A − L reach. This indicates that at a flow discharge of 4,000 m³/s, the equilibrium sediment concentrations are roughly 39.2 kg/m³, 36.4 kg/m³, 55.2 kg/m³, and 72.0 kg/m³, respectively. Using Eq. (27) along with the theoretical sediment delivery ratios for the four river reaches, the equilibrium sediment concentration—where erosion and aggradation are balanced—for the entire lower reach at a flow discharge of 4,000 m³/s is 48.6 kg/m³. This corresponds to an incoming sediment coefficient of 0.0122 kg∙s/m⁶. Li et al.^39^ proposed that the maximum flow discharge during flood events should not exceed the minimum bankfull flow discharge under non−floodplain conditions. The recommended optimal sediment concentration is 60 kg/m³, and the flow discharge ranges from 3500 to 4000 m³/s. Shen et al.^71^ demonstrated that when the flood flow discharge is 4000 m³/s and the river aggradation ratio ranges from 0% to 15%, the flood sediment concentration ranges from 50 kg/m³ to 75 kg/m³. Xu^69^ suggested that when the sediment delivery ratio ranges from 0.85 to 1.0, the flow discharge should be between 2600 and 4800 m³/s, while the sediment concentration should range from 30 to 50 kg/m³. Guo et al.^37^ suggested that the combination of 3000 m³/s < *Q*< 4000 m³/s and 20 kg/m³ <*S* < 60 kg/m³ in the T − H reach is regarded as the optimal configuration for sustained balanced sediment transport downstream. The aforementioned research findings are broadly consistent with the results obtained in this study, indicating that the theoretical expression established herein is reasonable.

### Impact of the width-to-depth ratio on the sediment delivery ratio

**Fig. 11:**
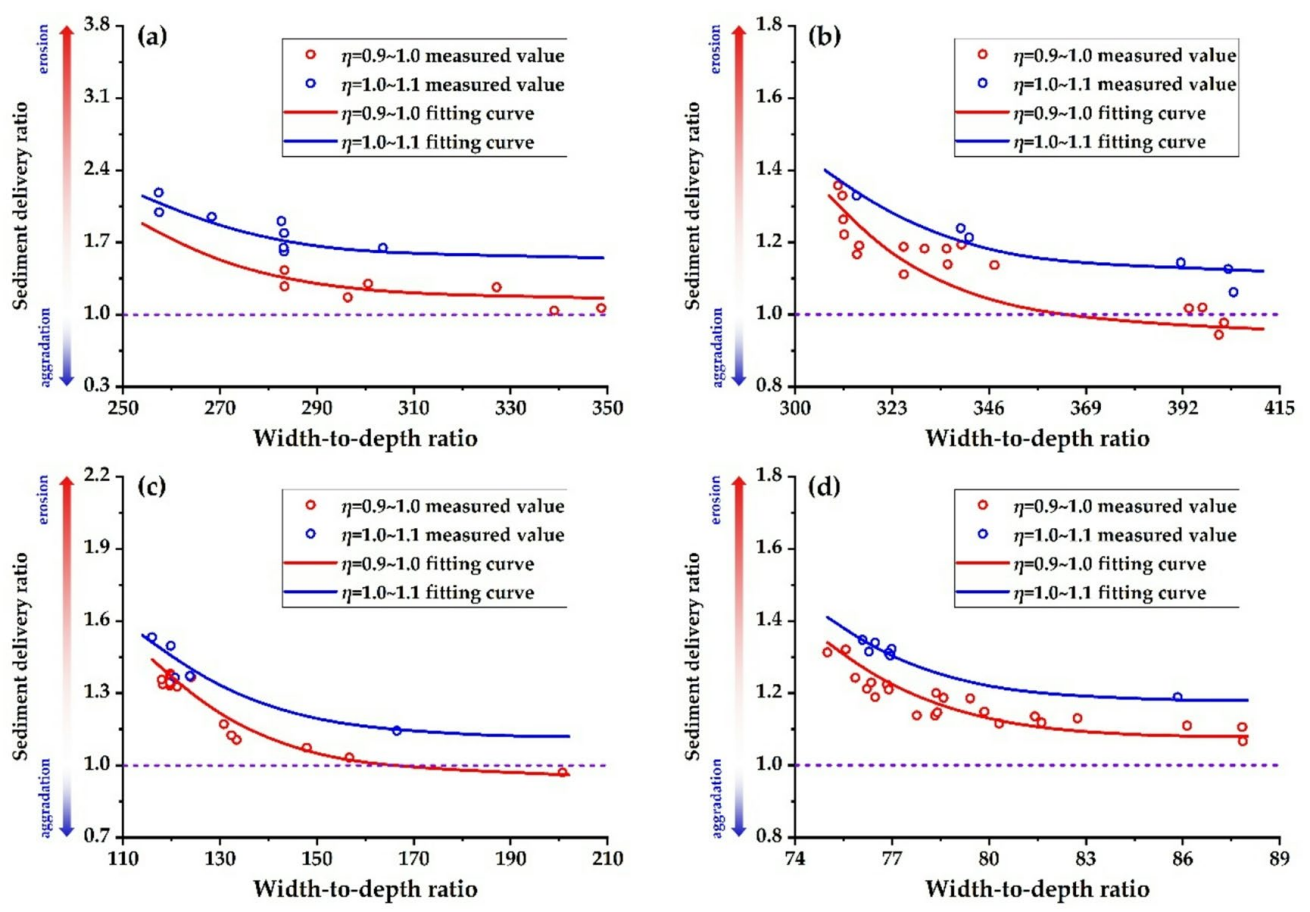
Relationship between sediment delivery ratio and width-to-depth ratio (incoming sediment coefficient is 0.005 ~ 0.01 kg ∙ s/m^6^). **(a)** T − H reach, **(b)** H − G reach, **(c)** G − A reach, **(d)** A − L reach.

Figure 11 illustrates the scatter plot of the sediment delivery ratio with the width-to-depth ratio. The graph shows that the sediment delivery ratio gradually decreases as the width-to-depth ratio increases, given the same water load variation coefficient. At the same time, a smaller water load variation coefficient leads to a smaller sediment delivery ratio.

**Fig. 12:**
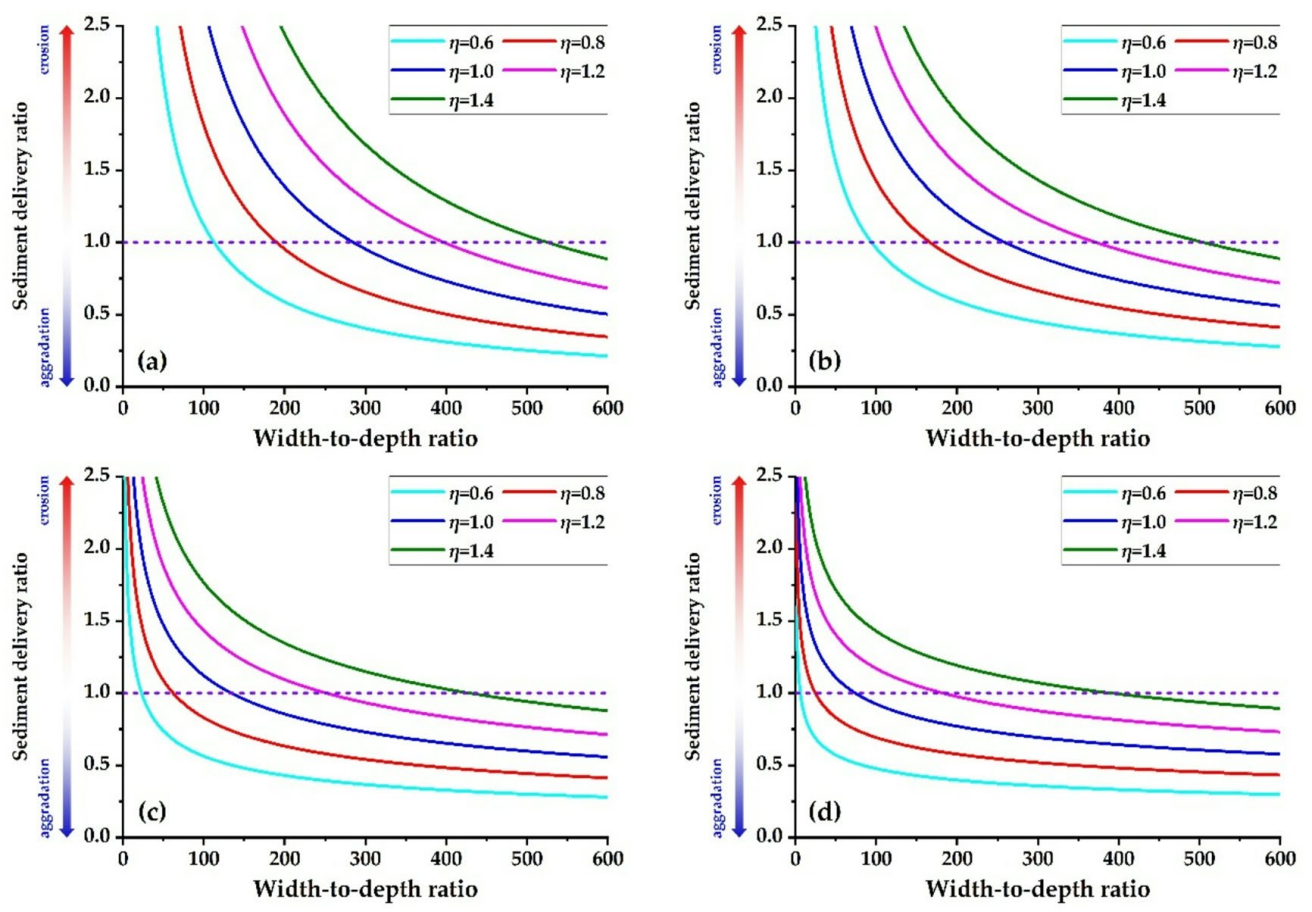
Relationship between beach width depth ratio and Sediment Delivery Ratio in the various reaches. **(a)** T − H reach, **(b)** H − G reach, **(c)** G − A reach, **(d)** A − L reach.

This study employs Eq. (19) to determine the relationship between the width-to-depth ratio and the sediment delivery ratio for each river reach, under a flow discharge of 4,000 m³/s and a sediment concentration of 60 kg/m³ (incoming sediment coefficient = 0.015 kg·s/m⁶), as illustrated in Fig. 12. Under the current boundary conditions regarding the median particle size of bed sediment and the river gradient, the sediment delivery ratio decreases as the width-to-depth ratio increases in the T − H reach, extending to the H − G, G − A, and A − L reaches. With the same width-to-depth ratio, as the water load variation coefficient increases, the sediment delivery ratio in each river reach will gradually rise.

**Fig. 13:**
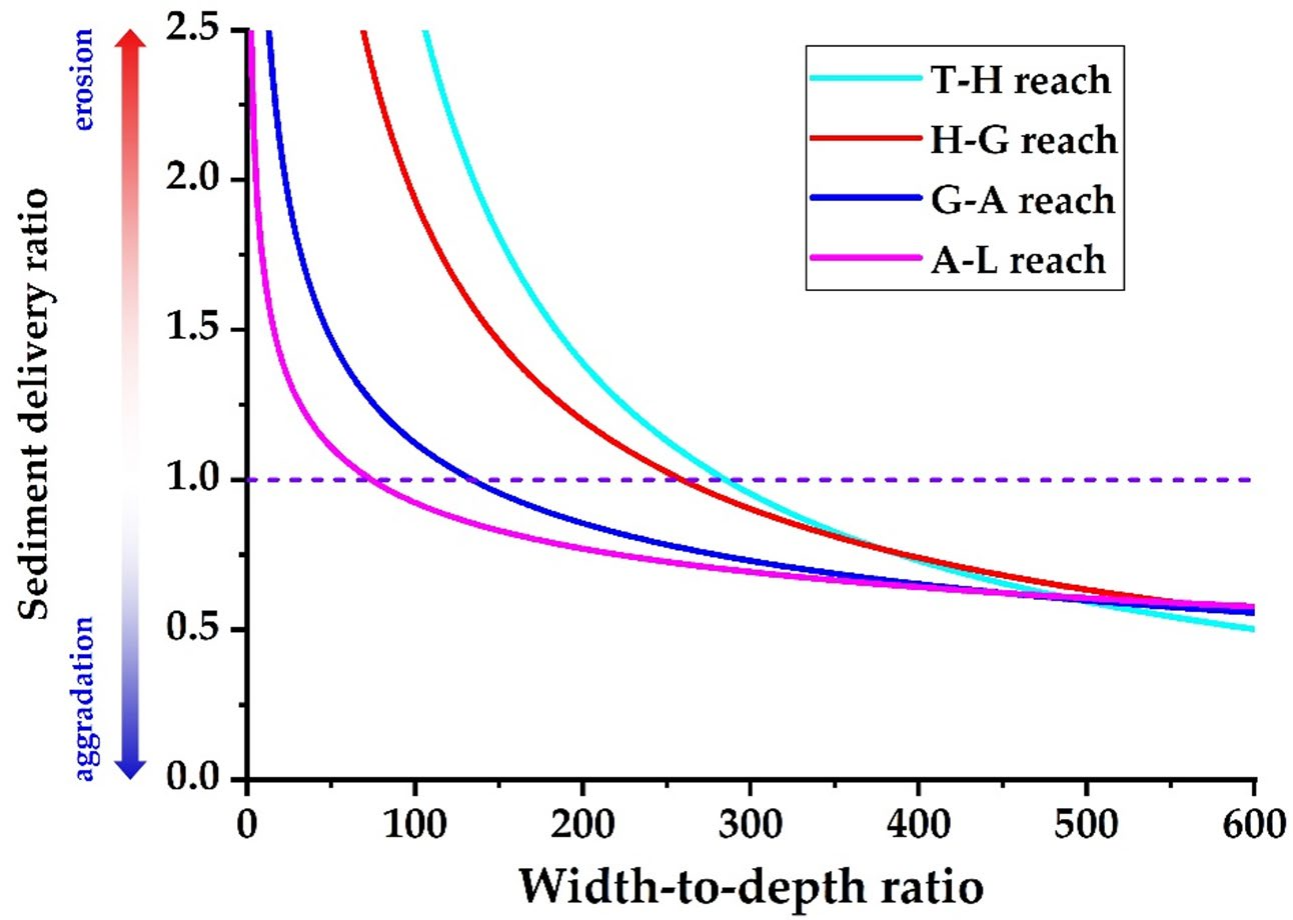
Relationship between width − to−depth ratio and sediment delivery ratio (*η* = 1.0).

Figure 13 illustrates the relationship between the width-to-depth ratio and the sediment delivery ratio for each river reach when the water load variation coefficient is 1.0. When the sediment delivery ratio of the river reach is 1.0 and the incoming sediment coefficient is 0.015 kg∙s/m^6^, the width-to-depth ratios in the T − H, H − G, G − A, and A − L reaches are 285, 259, 134, and 74, respectively. Currently, the bankfull width-to-depth ratios in the T − H, H − G, G − A, and A − L reaches are 344, 338, 143, and 60, respectively. This suggests that the river’s width should be further reduced in the reaches upstream of Gaocun to enhance the overall sediment transport capacity of the LYR.

## Conclusion

Based on hydrologic data and topographic data from 2000 to 2023 in the LYR, this paper analyzes the response of the sediment delivery ratio to water-sediment and riverbed boundary conditions during flood events. It elucidates the sediment transport characteristics of each river reach in the LYR under current boundary conditions. The main research conclusions are as follows:

(1) After 2000, the average flow discharge, sediment concentration, and sediment coefficient of flood events were focused in two distinct periods: 2000 − 2014 and 2018 − 2023, which relate to the operation mode of Xiaolangdi Reservoir. Since 2000, the water load variation coefficient in the T − L reach has exhibited a trend of initially increasing, followed by a decrease, and then increasing again, with the minimum water load variation coefficient reached in 2000.

(2) From 2000 to 2023, the bankfull width and depth in various river reaches showed an increasing trend, with the bankfull width expanding by about 1.5 times and the bankfull depth doubling. The overall trend of the bankfull width-to-depth ratio in each river reach is decreasing. After 2006, the bankfull width-to-depth ratio decreased significantly, indicating a trend of synchronous increase in both bankfull width and depth. The operation of the Xiaolangdi Reservoir has caused the bed sediment in the LYR to become coarser to varying extents, with the coarsening increasing closer to the dam. The river gradient upstream of Gaocun shows an increasing trend over time, while the river gradient downstream of Gaocun shows a decreasing trend over time.

(3) Factors such as the incoming sediment coefficient, the median particle size of bed sediment, and the width-to-depth ratio are negatively correlated with the sediment delivery ratio. In contrast, the water load variation coefficient and river gradient positively correlate with the sediment delivery ratio. Compared to considering only runoff and sediment conditions, the theoretical equation that includes the riverbed boundary provides a better fit to the measured sediment delivery ratio data, indicating that the riverbed boundary is a significant factor influencing the sediment delivery ratio.

(4) Under the current riverbed boundary conditions, when the sediment delivery ratio is equal to 1.0, the sediment transport coefficients for the T − H, H − G, G − A, and A − L reaches are 0.0098 kg∙s/m^6^, 0.0091 kg∙s/m^6^, 0.0138 kg∙s/m^6^, and 0.0180 kg∙s/m^6^, respectively, indicating that the A − L reach has the highest sediment transport capacity. To ensure that each river reach adapts to the same runoff and sediment conditions, the reach above Gaocun must continue to narrow its width and decrease the width-to-depth ratio. In contrast, the reach below Gaocun has already satisfied the requirements for this ratio.

## Data Availability

All the data and materials in the current study are available from the corresponding author upon reasonable request.
